# Seroprevalence of Dengue, Chikungunya, and Zika Viruses Among Febrile Patients in Dhaka, Bangladesh: A Hospital-Based Cross-Sectional Study

**DOI:** 10.3390/pathogens15010031

**Published:** 2025-12-25

**Authors:** Abir Dutta, Kazi Istiaque Sanin, Azizur Rahman Sharaque, Mahbub Elahi, Bharati Rani Roy, Md. Khaledul Hasan, Md. Sajjadur Rahman, Md. Shakil Ahamed, Mohammad Enayet Hossain, Md. Shafiqul Islam, Nuzhat Nadia, Goutam Kumar Dutta, Mohammed Ziaur Rahman, Md. Nasir Ahmed Khan, Md. Nazmul Islam, Fahmida Tofail

**Affiliations:** 1 International Centre for Diarrhoeal Disease Research, Bangladesh (icddr,b), GPO BOX 128, 68, Shaheed Tajuddin Ahmed Sarani, Dhaka 1212, Bangladesh; 2 Communicable Disease Control (CDC), Directorate General of Health Services, Ministry of Health and Family Welfare, Dhaka 1212, Bangladesh

**Keywords:** Dengue virus, Chikungunya virus, Zika virus, seroprevalence, arboviruses, Bangladesh, vector-borne diseases, public health surveillance, Aedes

## Abstract

Dengue (DENV), Chikungunya (CHIKV), and Zika (ZIKV) are emerging arboviral threats in Bangladesh, transmitted by Aedes mosquitoes thriving in urban Dhaka. Overlapping symptoms complicate diagnosis, and Bangladesh-specific data on arboviral antibody reactivity are limited. In four hospitals of Dhaka, we conducted a cross-sectional study on 438 febrile patients aged ≥10 years, collecting samples between September and December 2023 to describe arboviral antibody reactivity and their distribution across selected demographic and environmental characteristics. Rapid diagnostic tests (RDTs) for DENV and CHIKV were performed, followed by enzyme-linked immunosorbent assay (ELISA) on RDT-reactive samples. Participants had a mean age of 30 years (±13.5); two-thirds were male, and most lived in crowded, low-income households. RDTs indicated DENV/CHIKV antibody reactivity in 40% of participants; 170 samples underwent ELISA, suggesting DENV IgG reactivity in 33.5% and IgM reactivity in 15.5%. CHIKV IgG reactivity (0.7%) was low and ZIKV IgG was reactive in 21% of total samples, and IgM was reactive in one (0.2%); most ZIKV IgG-reactive samples also showed DENV IgG reactivity, suggesting cross-reactivity. DENV IgG and IgM reactivity were associated with lower education, while ZIKV IgM reactivity was associated with older age. Awareness of Aedes mosquitoes was low, and environmental risk factors were common. This study provides cross-sectional data on serological reactivity against DENV, CHIKV, and ZIKV among febrile patients attending four hospitals of Dhaka, without aiming to establish etiologic causes of illness. ZIKV IgG antibody reactivity requires confirmatory testing to distinguish true infections from other arboviral cross-reactivity. Strengthened community-based surveys, better public awareness, and sustained vector control are critical for reducing arboviral disease risks in urbanizing settings like Dhaka, Bangladesh.

## 1. Introduction

Arboviral infections, including Dengue, Chikungunya, and Zika, are arthropod-borne viral diseases mainly spread through *Aedes aegypti* and *Aedes albopictus* mosquitoes [1,2]. These mosquitoes remain active throughout the year, with populations typically surging during the rainy season (for example, from August to October) in many tropical regions [3]. Also, these mosquitoes have adapted remarkably to urban environments, thriving in various artificial water-holding containers, including vases, water tanks, discarded tires, and subterranean sites like septic tanks and construction sites [1].

Dengue, the most common mosquito-borne viral infection among the three, was first isolated in 1943 during an epidemic in Japan [4,5,6]. Dengue virus (DENV) is prevalent worldwide, putting an estimated 3.9 billion people, mostly in tropical and subtropical regions, at risk of infection [4]. Common clinical manifestations include severe headache, high fever (up to 40 °C/104 °F), retro-orbital pain, rash, swollen lymph nodes, nausea, vomiting, and intense joint and muscle pain, with more serious complications such as dengue hemorrhagic fever and dengue shock syndrome sometimes emerging after the fever subsides [5,6].

The Chikungunya virus (CHIKV) was initially recognized in Tanzania in 1952 and has been known for sporadic African and Asian outbreaks over subsequent decades [7]. However, in coastal Kenya, a large-scale outbreak in 2004 brought the virus to global attention and triggered widespread epidemics across the Indian Ocean islands and India [8,9]. Infection with CHIKV is generally marked by a sudden onset of fever accompanied by severe joint pain (arthralgia), headache, muscle pain, joint swelling, and rash; the joint pain may persist for months or even years in some patients [8].

The Zika virus (ZIKV) was initially identified in Uganda in 1947 from a rhesus monkey, and in 1954, the first reported human case was identified in Nigeria [10,11]. Although its primary transmission route is via mosquitoes, ZIKV can also spread via sexual contact, by blood transfusions, and from an infected mother to her unborn child [3]. Evidence shows approximately 80% of ZIKV infections are asymptomatic; when symptoms do occur, they tend to be mild, featuring fever, rash, joint pain, headache, and conjunctivitis, but the virus has also been related to serious problems such as microcephaly in newborns, acute myelitis, meningoencephalitis, and Guillain–Barré syndrome [3,12]. However, serological diagnosis is challenging due to cross-reactivity of antibodies elicited by antigenically closely related viruses, particularly DENV, ZIKV, West Nile, and other members of the Flavivirus genus [3].

Arboviral infections, such as DENV and ZIKV, trigger a characteristic immune response critical for interpreting serology. Immunoglobulin M (IgM) appears within 3–5 days, peaks at 7–14 days, and declines over weeks, while Immunoglobulin G (IgG) arises later and may persist for years, reflecting past or secondary infection. Secondary dengue infections can elicit strong IgG without a corresponding IgM rise, complicating interpretation in regions with co-circulating flaviviruses [13]. Cross-reactivity between DENV and ZIKV, due to shared envelope and membrane protein epitopes, can lead to false-positive results in ELISA or rapid diagnostic tests [14,15]. Neutralization assays, including plaque reduction neutralization tests (PRNT), remain the gold standard [16]. In practice, combined serological, molecular, and epidemiological approaches are recommended to improve diagnostic accuracy [17]. Understanding antibody kinetics and cross-reactivity is therefore essential for interpreting serological data in endemic settings.

In Bangladesh, the incidence of mosquito-borne viral diseases has risen markedly [18]. First reported in 1964, DENV has caused multiple outbreaks in Bangladesh over subsequent decades [19]. Notably, from January to December 2019, Dhaka city reported 51,179 hospitalized Dengue cases [20]. Bangladesh also reported its first Chikungunya outbreak in 2008, first ZIKV case in 2016, and experienced a significant Chikungunya outbreak between April and September 2017, resulting in around 13,800 documented Chikungunya instances in Dhaka alone [21,22,23]. Recent reports have identified multiple Zika cases in Dhaka, indicating co-transmission with DENV and CHIKV [24]. In Bangladesh, routine clinical diagnosis of dengue primarily relies on NS1 antigen detection and IgM/IgG antibody tests, which are widely available in both public and private healthcare facilities. PCR testing for dengue is performed mainly in specialized or reference laboratories and is not routinely used for all acute cases. For Chikungunya and Zika, routine antibody or PCR testing is not commonly performed in clinical settings. These tests are typically conducted only under specific circumstances, such as outbreak investigations, targeted surveillance, or research studies.

Due to the common vectors and similar clinical manifestations among DENV, CHIKV, and ZIKV, misdiagnosis is common; so, serological surveys describing antibody reactivity patterns can contribute to understanding exposure rates and identifying populations at higher risk. Southeast Asia has long been recognized as a hotbed for arboviral infections, frequently experiencing DENV and CHIKV outbreaks [2,3]. Rapid urbanization, high population densities, and favorable climatic circumstances in this region further facilitate the mosquito vector spread, driving recurring epidemics [2,3].

Although global seroprevalence studies for DENV, CHIKV, and ZIKV have been performed in high-population regions including Brazil, Nigeria, Indonesia, Thailand, and Ethiopia, comprehensive data describing antibody reactivity patterns of DENV, CHIKV, and ZIKV in urbanizing settings of Dhaka, Bangladesh remain limited [8,12].

Bangladesh continues to face recurrent outbreaks of arboviral diseases, with DENV representing the largest public health threat and CHIKV and ZIKV also documented in the region. Understanding how arboviral antibody patterns appear among patients seeking care can help inform and refine surveillance efforts in densely populated urban settings where transmission risk is high.

In this context, this study aimed to describe cross-sectional serological data on antibody reactivity patterns to DENV, CHIKV, and ZIKV among febrile patients attending urban tertiary hospitals in Dhaka, Bangladesh. Febrile patients were selected as a clinically relevant group from whom serological specimens are commonly collected, allowing description of antibody reactivity detectable at the time of sampling. It further sought to summarize how observed arbovirus-targeted antibody reactivity patterns were distributed across sociodemographic characteristics, environmental conditions, and personal protective practices. By exploring these factors, this study aimed to enhance epidemiological understanding and support surveillance-based public health interventions in urban settings.

## 2. Materials and Methods

### 2.1. Study Design

This hospital-based, cross-sectional study was conducted from September 2023 to June 2024. Data and blood samples were collected from September 2023 to December 2023 to describe antibody reactivity (IgM and IgG) to arboviral antigens (DENV, CHIKV, and ZIKV) among patients presenting with acute febrile illness. Laboratory testing and analysis continued through Jun 2024.

### 2.2. Study Population

The study population consisted of patients aged ≥10 years presenting with a history of acute fever (>38 °F/100.4 °C) confirmed either through direct temperature measurement at enrollment or recent clinical records lasting at least five days prior to coming to hospital, with or without persistence of fever at enrollment and recruited from both outpatient and inpatient departments of selected health facilities. Pregnant women, terminally ill patients, those in acute emergency conditions, and individuals unable or unwilling to provide informed consent were excluded from the study. Blood samples were collected during the acute phase of illness, generally between days 5 and 7 after fever onset.

### 2.3. Study Setting

This study was conducted in Dhaka, Bangladesh, across four public tertiary-level hospitals:

- Shaheed Suhrawardy Medical College Hospital
- DNCC Dedicated COVID-19 Hospital
- Kurmitola General Hospital
- Kuwait Bangladesh Friendship Government Hospital

These hospitals were strategically selected to ensure a geographically and demographically representative sample of febrile patients, reflecting diverse urban populations within Dhaka.

### 2.4. Sample Size

The sample size was calculated using the Cochran formula for cross-sectional studies (*n* = Z^2^p(1 − p)/d^2^), assuming a 95% confidence level (Z = 1.96), a 5% margin of error (d = 0.05), and an assumed prevalence (p) of 0.5 to yield the maximum possible sample size given the lack of prior estimates for combined arboviral seroprevalence among febrile patients in Dhaka. This initial calculation resulted in a requirement of approximately 385 participants. To account for potential non-response or attrition, estimated at 12%, the sample size was increased accordingly. After adjusting for this expected loss, the final target sample size was approximately 438 participants.

In this hospital-based context, convenience sampling was the most feasible approach to recruit participants presenting with acute febrile illness. Given the limited timeframe and the need to enroll patients as they naturally appeared in outpatient and inpatient departments, recruiting by convenience allowed for efficient data collection under real-world conditions. The study received ethical approval from the icddr,b Research Review Committee (RRC) and Ethical Review Committee (ERC), and written informed consent was obtained from all participants before enrollment.

### 2.5. Data and Sample Collection

**Figure 1:**
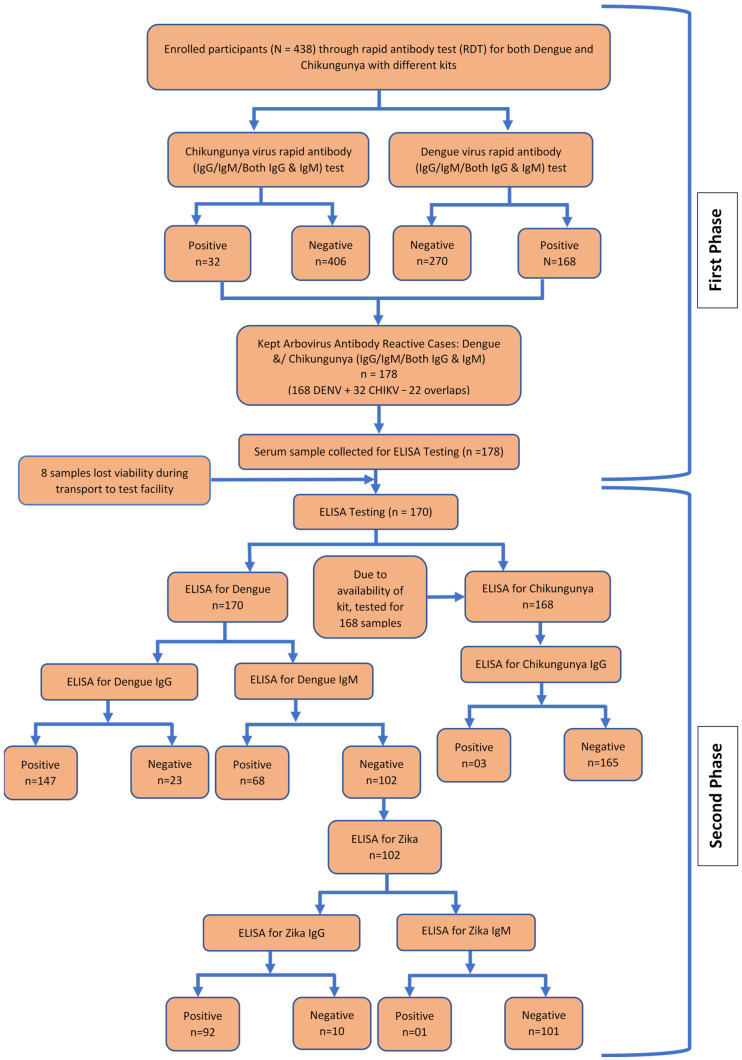
Study Flow Diagram.

We collected data from September 2023 to December 2023 using semi-structured, interviewer-administered questionnaires combined with blood sample analysis. Subsequent analyses of collected samples were conducted up to June 2024. This hospital-based approach was intended to identify potential arboviral antibody reactivity patterns among healthcare-seeking febrile patients in a high-risk setting like Dhaka, while acknowledging that other infectious causes may also have contributed to febrile presentations. The questionnaire covered socio-demographic factors, environmental exposures, clinical symptoms, and history of prior clinically suspected arboviral illness (self-reported), recognizing that laboratory confirmation was not available. Trained data collectors administered the questionnaire using tablet-based electronic forms, which were completed immediately after the primary screening assessment. Clinical history prior to hospital presentation was obtained from patient or caregiver interviews and verified through referral or outpatient records when available, which documented previous temperature measurements or medical notes. All data were verified by trained research physicians at enrollment. Figure 1 depicts the study flow diagram that we followed for sample collection and testing. Rapid diagnostic tests (RDTs) were performed using fingerstick blood samples to check IgM and IgG antibody reactivity for only DENV and CHIKV following the manufacturers’ standard protocols provided inside the box of RDTs. Each cassette was labeled with a unique participant ID, and results were interpreted visually within 15–20 min. We used qDetect^TM^ Dengue IgG/IgM Combo Test Device (Q03-02a-22) and qDetect^TM^ Chikungunya IgG/IgM Combo Test Device (Q03-09-42) manufactured locally by OMC HealthCare, Dhaka, Bangladesh. According to the documentation provided inbox by the manufacturer, the Dengue RDT demonstrates IgM and IgG sensitivities of 97.4% and 98.6%, respectively, with a specificity of 99%, while the Chikungunya RDT shows a sensitivity of 95.8% and specificity of 97.0%. No serum dilutions for DENV and CHIKV RDTs were required as per the manufacturer’s guidelines. ZIKV RDTs were unavailable due to import limitations. Positive RDT results were further tested via a competitive enzyme-linked immunosorbent assay (ELISA) protocol for DENV, CHIKV, and ZIKV antibody reactivity. From each participant whose sample reacted positively on rapid tests at the hospital after interview, about 3–5 mL of venous blood into a plain Vacutainer tube was taken. Serum was then obtained by centrifuging the samples at 3000 rpm for 10 min. Into sterile cryovials, aliquots of 0.5–1 mL were transferred, labeled with unique identification codes, and promptly transported to the laboratory, where they were stored in an ultralow freezer (−80 °C) until ELISA analysis.

Both IgG and IgM for DENV and ZIKV, and only IgG for CHIKV (due to resource unavailability), were assessed using ELISA. RDTs were employed as an initial screening tool to identify probable arboviral reactivity at point-of-care and to guide subsequent ELISA testing. The combined use of RDT and ELISA improved logistical feasibility in the field and ensured that laboratory resources were efficiently used for more elaborate testing of samples with initial antibody reactivity.

For DENV and ZIKV, serum IgG and IgM antibodies were measured using commercial ELISA kits (Vircell S.L., Granada, Spain; Dengue IgG: G1018, Dengue IgM: M1018, Zika IgG: G1023, Zika IgM: M1023) [25,26]. The sensitivity and specificity of the ELISA test kits used were reported by the manufacturer to be 98% and 100% for anti-dengue IgG, 98% and 99% for anti-dengue IgM, 90% and 99% for anti-Zika IgG, and 96% and 97% for anti-Zika IgM, respectively [25,26]. Dengue IgG and Zika IgG/IgM antibody detections were performed as indirect ELISAs (IgG sorbent for IgM applied directly in wells), while Dengue IgM antibody was tested using the μ-capture format. Serum was diluted 1:21 for IgG assays and 1:11 for the μ-capture IgM antibody assay following the manufacturer’s instructions. Negative, cut-off, and positive controls were included in each run, plates were read at 450/620 nm, and results were expressed as an Antibody Index = [Optical Density (OD) of sample/mean OD cut-off] × 10, with <9 negative, 9–11 equivocal, and >11 positive. ZIKV ELISA testing was performed only on samples that were negative for DENV IgM to reduce the likelihood of cross-reactive acute-phase antibody responses. We note that DENV IgG antibodies can also cross-react with ZIKV antigens; therefore, this approach reduces but does not eliminate cross-reactivity. Serum IgG antibodies to CHIKV were tested using a commercial ELISA kit (MyBioSource, San Diego, CA, USA: MBS1610578) based on a sandwich principle, following the manufacturer’s instructions for sample processing, incubation, washing, and colorimetric detection at 450 nm [27]. Results were interpreted relative to kit-defined criteria, with the IgG cut-off calculated as the mean OD of negative controls + 0.15, OD ≥ 1.00 considered positive, and OD ≤ 0.10 considered negative.

Data were collected using tablet-based forms and entered daily into a central KOBO Collect server for subsequent analysis.

### 2.6. Study Definitions

Acute febrile illness: Defined as fever ≥38 °C/100.4 °C of sudden onset, lasting ≥5 days prior to seeking healthcare service, with or without accompanying nonspecific symptoms such as headache, myalgia, rash, or arthralgia, and without a confirmed alternative diagnosis.

Seroprevalence: The proportion of participants in the clinical (febrile) study population with detectable antibodies (IgM or IgG) against Dengue, Chikungunya, or Zika viruses. In this study, the term “seroprevalence” refers strictly to antibody reactivity within the enrolled febrile cohort and does not represent population-level prevalence.

Rapid Diagnostic Test (RDT): A membrane-based immunoassay to detect IgM and IgG antibodies qualitatively.

ELISA (Competitive): A suggestive test for antibody presence using enzyme-linked immunosorbent assays, employed for participants testing positive by RDT.

### 2.7. Variables

Serological Variables: Results of RDT and ELISA testing for the reactivity of IgM and IgG antibodies to DENV, CHIKV, and ZIKV.

Demographic Variables: Age, sex, education level, occupation, and socio-economic status.

Environmental Variables: Water storage practices, drainage conditions, waste management, and history of public health interventions.

Clinical Variables: Symptoms such as fever, joint pain, rashes, and travel history.

### 2.8. Data Analysis

We calculated descriptive statistics, including frequencies, percentages, means, and standard deviations, to summarize the demographic and clinical data. Associations between categorical variables were examined through cross-tabulations, and statistical significance was assessed using Chi-square tests, Fisher’s exact tests, and independent sample t-tests, as appropriate. These exploratory analyses reflect patterns of antibody reactivity only and do not indicate confirmed infection or causal relationships. For the combined arbovirus association analysis, participants with any positive ELISA antibody reactivity for DENV, CHIKV, or ZIKV (IgG and/or IgM) were recoded as ‘antibody-reactive’ for descriptive subgroup analyses. For descriptive subgroup analyses, participants without ELISA antibody reactivity (and those not tested by ELISA) were used as an internal comparison group within the febrile cohort; these are not population ‘non-cases’ and do not represent confirmed absence of infection. All comparative analyses are exploratory and limited to the healthcare-seeking sample. We considered a *p*-value of <0.05 as statistically significant and set the confidence intervals at 95%. Data were visualized using bar charts where relevant. Data were managed and analyzed using statistical software R version 4.3.3 and RStudio 2023.12.1.

Because this study was designed to document antibody reactivity patterns among febrile patients presenting to health facilities, no asymptomatic or non-febrile comparison group (“non-cases”) was included. The absence of a non-case group reflects the diagnostic, hospital-based nature of the investigation and precludes population-level seroprevalence estimation. Therefore, analytical comparisons were restricted to variations within the febrile cohort only.

## 3. Results

This cross-sectional study was conducted in selected healthcare facilities in Dhaka, Bangladesh, among 438 febrile individuals ranging from 10 to 80 years of age. Among the four study sites, one-third (33.8%, *n* = 148) of the participants were from Shaheed Suhrawardy Medical College Hospital, followed by DNCC Dedicated COVID-19 Hospital (25.8%, *n* = 113), Kurmitola General Hospital (25.3%, *n* = 111), and Kuwait Bangladesh Friendship Government Hospital (15.1%, *n* = 66). The variation in participant distribution across the four study sites was likely due to recruiting by convenience and the differing patient flow of febrile cases at each hospital.

### 3.1. Sociodemographic Descriptions of Participants

The study included 438 participants (Table 1), with a mean age of 30 years (±13.5). Two-thirds of the participants were male (66.9%), and a slight majority were currently married (54.7%). A substantial proportion had either no formal education or had only completed primary education (55.9%). Approximately 58% were employed, while 21.7% were students and 16% were housewives. Most participants (97.5%) identified as Muslim. Household crowding was common, with nearly 70% residing in households with ≥2 persons per room. The majority reported a family monthly income of ≤30,000 BDT (69.4%).

### 3.2. General Awareness About Aedes Mosquitoes and the Surrounding Physical Environment of the Participants, Protective Measures, Disease History, Symptoms, and Health Information Sources

Knowledge of Aedes mosquitoes was notably low: only 40.6% correctly identified Aedes as the vector for dengue, Zika, and chikungunya, while 58.7% responded “don’t know” (Table 2). Understanding of mosquito biting times and breeding habitats was also limited. Only 13.2% correctly identified clean water as the primary breeding site, while 76.3% incorrectly chose wastewater.

Environmental risk factors were prevalent: 30.7% lived near open drains, 35.3% near construction sites, and 24% near ongoing road or bridge work. Notably, 23.7% reported the presence of potential Aedes breeding items near their homes. Despite this, 98.6% acknowledged recent municipal cleanliness or drainage activities.

Regarding personal protection, mosquito nets (84.7%) and coils (74.7%) were the most frequently reported measures. Less than 15% used repellents, sprays, or practiced environmental cleanliness. A negligible proportion (1.1%) reported using no protective measure.

Recent travel outside Dhaka was reported by 12.1% of participants. A past diagnosis of dengue was reported by 5.7%, chikungunya by 1.4%, and none reported Zika. Fatigue (95.9%), headache (87.2%), and fever (86.2%) were the most common current symptoms.

Media (66.4%) and family/friends (72.6%) were the leading sources of health information, followed by health workers (42%) and government campaigns (24.9%).

### 3.3. Arbovirus Antibody Reactivity

**Figure 2:**
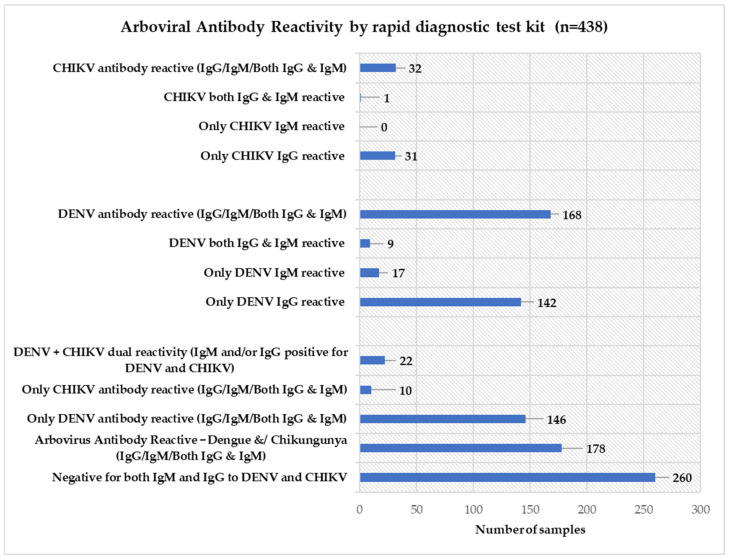
Number of participants reactive for arbovirus (DENV and CHIKV) antibody in serum evaluated by rapid diagnostic test kit (*n* = 438).

Figure 2 shows the distribution of DENV and CHIKV antibody presence suggested by rapid diagnostic tests (RDTs) among the 438 samples. Overall, 178 (40.6%) were positive for at least one arboviral antibody (IgG and/or IgM). DENV antibodies were detected in 168 (38.4%) participants, including 142 (32.4%) with only IgG antibody, 17 (3.9%) with only IgM antibody, and 9 (2.1%) with both. CHIKV antibodies were detected in 32 (7.3%) participants, primarily only IgG antibody in 31 (7.1%), with only no case each of only IgM antibody and one case of dual IgM/IgG antibody positivity. Dual DENV + CHIKV positivity was identified in 22 (5.0%) participants. The predominance of IgG antibody over IgM antibody indicates that most participants had suggestive data of past rather than acute infection.

Among the RDT-reactive participants, 178 samples were identified for ELISA testing; however, 8 samples were lost and could not be tested, leaving 170 samples available for ELISA analysis. Table 3 shows that, among these 170 samples, 147 were reactive for DENV IgG and 68 for DENV IgM. When expressed relative to the total study population (*n* = 438), this corresponds to 33.6% and 15.5%, respectively.

For ZIKV ELISA testing, 102 participants who showed negative DENV IgM antibody reactivity on ELISA were tested. A large proportion, 90.2% (*n* = 92) of tested samples, equivalent to 21.0% of the overall study population, were reactive for ZIKV IgG antibodies. However, only one individual (1.0%) had a positive ZIKV IgM antibody reactivity.

For CHIKV, 168 participants were tested with ELISA. Of them, 1.8% (*n* = 03) showed positive reactivity for CHIKV IgG antibody, which accounted for 0.7% of the total study population. Due to unavailability of a Chikungunya IgM testing kit, we could only test for IgG antibody.

Among the 26 RDT positive for DENV IgM antibody, 17 were positively reactive in ELISA for DENV IgM antibody. For DENV IgG antibody, out of 151 RDT positives, 140 were positive based on ELISA. For CHIKV IgG antibody, out of 32 RDT positives, only 1 was positive based on ELISA.

### 3.4. Combined Distribution and Overlap of Arboviral Seropositivity

**Figure 3:**
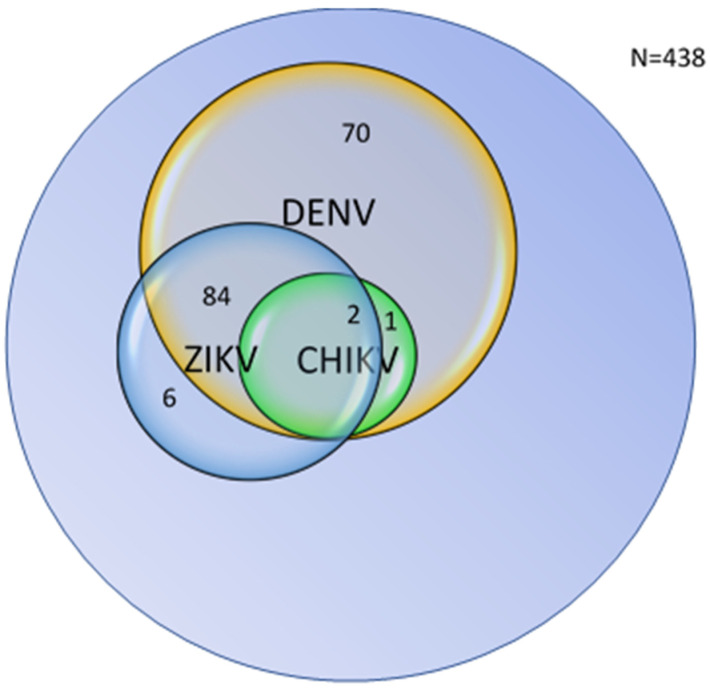
ELISA inter-test overlap (IgG and/or IgM for DENV, CHIKV, and ZIKV).

Table 4 and Figure 3 show the combined distribution of ELISA results for DENV, ZIKV, and CHIKV antibodies among 438 febrile patients. Among all participants, 62.8% were seronegative for all three arboviruses, whereas 37.2% showed positive reactivity for at least one virus.

DENV-only positive reactivity (IgM and/or IgG) accounted for 15.9% of participants, representing the largest single-virus group. ZIKV-only positive reactivity was detected in 1.4% of participants, while no participants were exclusively positive for CHIKV antibodies.

Dual or multiple antibody positivity was also observed. DENV + ZIKV dual positivity was the most common overlap, present in 19.2% of the cohort. A single participant (0.2%) showed dual DENV + CHIKV positivity, while two cases (0.5%) demonstrated triple-virus positivity for DENV, ZIKV, and CHIKV. No participants were positive for ZIKV + CHIKV only. Given the known antigenic cross-reactivity among flaviviruses, ELISA results are interpreted as suggestive rather than definitive virus-specific confirmation.

### 3.5. Exploratory Associations Between Sociodemographic and Environmental Factors with Arbovirus Seropositivity by ELISA

#### 3.5.1. DENV IgG Antibody Reactivity

Among the 438 participants, 147 (33.6%) tested positive for DENV IgG antibodies (Table 5). Education level was likely associated with DENV IgG antibody seropositivity (*p* = 0.051). Participants with lower education (no formal or primary incomplete) were more likely to show antibody reactivity, whereas those with higher secondary or tertiary education were more common in the non-reactive group. No significant associations were observed with age (*p* = 0.095), sex (*p* = 0.886), crowding index, or environmental exposures such as open drains or construction sites. Use of mosquito nets was almost equal for both seropositive and seronegative groups (84.4% vs. 84.9%, *p* = 0.885).

#### 3.5.2. DENV IgM Antibody Reactivity

Among the 438 participants, 68 (15.5%) positively reacted for DENV IgM antibody based on ELISA (Table 6). Lower education level (*p* = 0.022) appeared more common among participants with IgM reactivity, although this reflects antibody detection only and not confirmed or recent infection. No other factors, including age, sex, household crowding, or environmental risks, showed statistically significant associations with IgM seropositivity. Protective behaviors also did not differ significantly.

#### 3.5.3. ZIKV IgG Antibody Reactivity

Out of 102 participants tested, 92 (21% of total study population) were positively reactive for ZIKV IgG antibody (Table 7). ZIKV IgG ELISA reactivity (*p* = 0.005) appeared more frequently in older individuals (33.5 ± 15.0 years vs. 29.1 ± 13.0 years), though this does not reflect virus-specific exposure. No statistically significant associations were found between seropositivity and sex, education, environmental exposures, or protective behaviors.

#### 3.5.4. ZIKV IgM and CHIKV IgG Antibody Reactivity

Zika IgM antibody reactivity was found in 0.2% of the samples tested, representing a single participant. Seropositivity for Chikungunya IgG antibody reactivity was 0.7%, representing only three participants. Due to lack of variability, we were unable to look into the association between Chikungunya IgG and Zika IgM antibodies with other factors.

#### 3.5.5. Overall Arboviral Antibody Reactivity

Considering all arboviruses together (Table 8), 37.2% of participants showed antibody reactivity to at least one virus. No statistically notable patterns were observed between overall antibody reactivity and demographic or environmental variables.

## 4. Discussion

Our hospital-based cross-sectional study, conducted during the post-monsoon period in Dhaka, explored antibody reactivity to DENV, CHIKV, and ZIKV among febrile patients attending four urban tertiary care hospitals. This study further examined how observed antibody reactivity patterns of arboviruses varied across sociodemographic characteristics, environmental conditions, and personal protective practices.

Rapid diagnostic tests (RDTs) indicated IgM and/or IgG reactivity to DENV or CHIKV in more than two-fifths (40.6%) of participants, with DENV reactivity (38.4%) more common than CHIKV reactivity (7.3%). Dual DENV + CHIKV reactivity was seen in 5.0% of participants based on RDT.

ELISA testing provided further insight into the antibody reactivity patterns. Approximately 33.6% of the total samples showed DENV IgG antibody reactivity based on ELISA, while 15.5% showed DENV IgM antibody reactivity. These findings reflect patterns of serological reactivity rather than confirmed DENV infection. Although the IgG and IgM levels differ from those reported in a 2012 community-based survey in Dhaka [28], methodological and temporal differences limit direct comparison. The observed IgM reactivity indicates the presence of detectable arboviral antibodies in this cohort during the post-monsoon period, without implying a causal or temporal link to the monsoon season when vector density typically peaks [29].

Among the 32 participants who showed CHIKV antibody reactivity based on RDT, only 1 demonstrated IgG antibody reactivity based on ELISA. This discrepancy may reflect false positive RDT readings, or cross-reactivity, or the inherently different performance characteristics of the two assays. In our study, CHIKV IgG antibody reactivity based on ELISA was 0.7% (3 out of 438 cases), which was lower than the 2.4% plaque reduction neutralization test (PRNT) confirmed seroprevalence reported in a pre-2017 national survey [30].

In our study, ZIKV ELISA testing was performed on 102 samples that were negative for Dengue IgM antibody ELISA reactivity (a subset of those RDT positive for arboviral antibodies) to reduce but not eliminate the likelihood of detecting overlapping IgM reactivity. Of these, 90.2% showed ZIKV IgG reactivity, equivalent to 21.0% of the total samples, while only one participant (0.2%) showed ZIKV IgM reactivity. This ZIKV IgG antibody reactivity appears within the global reports from endemic settings, which the CDC-Atlanta places at approximately 16–23% [3,31], slightly below the World Health Organization’s Southeast Asia region estimates of around 22.8%, and higher than Thailand reporting of nearly 16% [6]. However, 86 of the 92 ZIKV IgG antibody reactive cases (93.5%) were also DENV IgG antibody reactive, leaving only 6 (6.5%) with ZIKV-only IgG antibody reactivity, equivalent to 1.4% of the total cohort. Given the strong serological overlap (as shown in Table 4), the presence of high ZIKV IgG antibody reactivity is probably due to flaviviral cross-reactivity rather than independent infection, echoing findings from other endemic regions [3,32,33,34].

Combined analysis of ELISA results suggested that 37.2% of participants showed antibody reactivity to at least one of the three arboviruses, while 62.8% were nonreactive for all. The most common pattern was DENV-only reactivity (15.9%), followed by dual DENV + ZIKV reactivity (19.2%), and only 1.4% were ZIKV-only reactive. Dual DENV + CHIKV (0.2%) and triple-virus reactivity (0.5%) were found in very few cases. These findings point toward significant flavivirus cross-reactivity, especially between DENV and ZIKV rather than simultaneous or sequential infections. Similar patterns have been observed in studies from Thailand and Brazil, where high dual-positivity rates could not be distinguished without confirmatory neutralization testing [32,33,34].

It is important to note that ELISA-based immunoassays cannot distinguish virus type-specific antibodies among antigenically and genetically related flaviviruses such as DENV and ZIKV. Therefore, the ELISA results were interpreted as indicative of antibody reactivity rather than definitive confirmation of virus-specific infection. Neutralization assays (e.g., plaque reduction neutralization test), which are considered the gold standard for confirming virus type-specific antibodies, were not performed due to financial and logistical constraints during the study period. The absence of confirmatory neutralization assays (e.g., PRNT) in this study limits our ability to definitively identify virus-specific antibodies. Prior research strongly recommends the use of such assays in flavivirus-endemic regions to differentiate true infections from cross-reactive immune responses [32,33,34]. Hence, while our data suggest possible ZIKV ELISA reactivity in Dhaka, these results should be interpreted cautiously. Future work incorporating molecular testing and neutralization assay validation is essential to establish whether ZIKV is genuinely circulating or if the findings reflect antibody cross-reactivity within the flavivirus group.

For the sociodemographic analysis, lower educational attainment showed borderline association with DENV IgG reactivity (*p* = 0.051) and was significantly associated with DENV IgM reactivity (*p* = 0.022). These patterns may reflect differential exposure or prevention practices, though causality cannot be inferred from cross-sectional data. Comparable associations have been reported in studies from other South and Southeast Asian countries [35].

ZIKV IgG antibody reactivity was more common among older participants (mean age 33.5 years vs. 29.1 years; *p* = 0.005), suggesting age-related variation in ELISA reactivity, though not interpretable as age-related exposure. This pattern likely reflects repeated flavivirus reactivity rather than ZIKV-specific antibody reactivity. Comparable age-related increases have been documented in long-term dengue-endemic populations [36,37].

Other variables like sex, household crowding, environmental exposures, and protective behavior did not show associations with DENV IgG or IgM antibody reactivity. This may be because dengue is highly common in the study area, so most people are exposed regardless of these characteristics [4]. Since IgG antibody reflects past infection, cross-sectional data may not match current behaviors or exposures [38]. Self-reported protective practices can also be affected by recall errors or over-reporting, which makes it harder to see real effects [39]. In addition, larger community factors such as mosquito density, local environment, and circulating virus types may play a stronger role than individual factors [40]. For the overall reactivity of arboviruses based on ELISA, no variable showed any significant association. Also, due to lack of variability association between Chikungunya IgG antibody and Zika IgM antibody, reactivity with other factors could not be seen.

Symptoms such as fatigue, headache, fever, myalgia, and arthralgia mirror the clinical overlap reported between dengue and chikungunya during previous outbreaks in Dhaka, Bangladesh in similar hospital settings and other countries [41,42]. Although all enrolled participants had a history of fever, 86.2% were febrile at the time of hospital assessment; some participants had received antipyretics or other treatment prior to presentation, or their fever had subsided by enrollment, and fever history was captured through interview and available referral records. Also, the frequent use of mosquito nets (84.7%) and coils (74.7%) highlights a reliance on nocturnal protection, which may stem from misconceptions that associate mosquito bites primarily with night, even though Aedes mosquitoes bite during the day [43,44]. Knowledge of mosquitos was moderate, with only 40.6% correctly identifying the mosquito vector and just 13.2% recognizing clean water as a breeding site, a gap also reported in other community surveys conducted in Western Jamaica, Nepal, and Bangladesh [45,46,47]. These findings underscore the need for targeted health education programs that emphasize the role of daytime protection (such as repellents, full-sleeve clothing, and decrease of breeding sites) and to remove negative beliefs as previously demonstrated by Banik and colleagues in a study of a rural area of Bangladesh and also by Tanvir Abir and colleagues in a study in Dhaka, Bangladesh [48].

Approximately one-third of participants reported living near open drains or construction sites, both recognized breeding grounds for vector mosquitoes [36]. However, no statistical differences in antibody reactivity were observed across these environmental factors, probably because in crowded urban areas, most people are exposed, so these factors do not strongly distinguish risk [4]. The continued arboviral antibody reactivity despite recent municipal cleanliness campaigns also suggests that standard vector control measures have limited long-term effectiveness, as reported in a systematic review and meta-analysis by Bowman and colleagues [38].

This study had several methodological strengths. Sampling across four tertiary hospitals captured data of febrile patients from diverse urban areas of Dhaka, enhancing representation within the care-seeking population. The combined use of rapid diagnostic tests (RDTs) and ELISAs provided a practical, multi-step serological approach that enabled preliminary characterization of arboviral antibody reactivity patterns. Integration of sociodemographic, environmental, and behavioral information allowed for an exploratory assessment of potential risk factors, while electronic data capture with daily validation reduced errors and improved data reliability. Importantly, this study provides serological data describing patterns of antibody reactivity to DENV, CHIKV, and ZIKV among urban febrile patients sampled in four hospitals of Dhaka, Bangladesh.

However, several limitations should be acknowledged. First, the loss of eight serum samples during transport underscores the need for stricter adherence to specimen handling and preservation protocols. Second, the unavailability of CHIKV IgM antibody ELISA kits and ZIKV RDTs hindered the accurate detection of acute-phase infections, primarily due to resource constraints. Third, the high ZIKV IgG antibody reactivity observed may largely reflect cross-reactivity with other flavivirus antibodies, as confirmatory neutralization assays such as PRNT or other neutralization tests were not performed. Although ZIKV testing was intentionally restricted to DENV ELISA IgM antibody non-reactive samples to reduce potential cross-reactivity, this selection approach may have introduced bias, limited comparability across groups, and may have overlooked ZIKV IgG antibody reactivity among DENV/RDT non-reactive participants. Moreover, the analysis was conducted on a subgroup with high DENV antibody reactivity, which may further increase the likelihood of DENV/ZIKV cross-reactivity and limit the generalizability of ZIKV IgG antibody reactivity findings to the broader cohort. In addition, testing for other flaviviruses such as West Nile or Usutu virus could not be undertaken due to limited resources. Fifth, the use of a hospital-based convenience sample of febrile patients restricts the generalizability of the findings to the wider community, as a considerable proportion of arboviral infections remain asymptomatic. Thus, the results primarily represent antibody reactivity among symptomatic healthcare seekers rather than population-level estimates. Sixth, only serological assays were performed, and molecular confirmation (e.g., RT-PCR for viral RNA) was not feasible, limiting verification of serological results. Also, while fever history was used for inclusion, samples were collected during the late acute or early convalescent phase, which could influence antibody detection. Finally, respondent and recall biases may have affected sociodemographic and environmental information, and the severity of reported symptoms was not comprehensively evaluated.

## 5. Conclusions

This study provides cross-sectional data on IgM and IgG antibody reactivity to DENV, CHIKV, and ZIKV among urban febrile patients in Dhaka, Bangladesh, a setting where co-circulation of these arboviruses has previously been documented. Within this context, our findings should be interpreted as reflecting patterns of arboviral serological reactivity rather than as confirmation of virus-specific infection, given the absence of confirmatory molecular or neutralization assays. In particular, the observed ZIKV antibody reactivity may reflect cross-reactive responses to DENV or other flaviviruses, rather than confirmed ZIKV infection. The presence ZIKV IgG antibody reactivity underscores the need for future studies incorporating virus-specific neutralization testing and molecular diagnostics to clarify the true extent of ZIKV circulation. The high proportion of seronegative febrile cases in our cohort suggests that a wide range of other infectious etiologies is probably contributing to the burden of acute undifferentiated fever. This finding reinforces the importance of integrating molecular diagnostics and surveillance in both hospital and public health settings to more accurately characterize febrile illnesses in Bangladesh. Community-based serosurveys, alongside facility-based research, will be important to capture population-level patterns of arboviral exposure over time. Consistent with prior surveys in Bangladesh, many participants were unaware of basic *Aedes aegypti* biology, highlighting common gaps in vector awareness that may impact preventive practices [47]. Addressing these gaps through sustained, context-specific education and prevention campaigns alongside continuous vector control rather than short-term clean-up efforts will be essential for reducing the risk posed by DENV, CHIKV, ZIKV, and other Aedes-borne infections in rapidly urbanizing cities like Dhaka.

## Figures and Tables

**Table 1:** Sociodemographic Description of the participants (*n* = 438).

Sociodemographic Description	
Age in years	30 (±13.5)
Sex	
Male	293 (66.9%)
Female	145 (33.1%)
Marital Status	
Never married/Single	180 (41.1%)
Currently Married	239 (54.7%)
Formerly Married (Separated/Divorced/Widowed)	18 (4.1%)
Educational Status	
No formal education/Primary incomplete	125 (28.54%)
Primary complete	120 (27.4%)
Secondary complete	60 (13.7%)
Higher	133 (30.36%)
Religion	
Muslim	427 (97.49%)
Other	11 (2.51%)
Occupation	
Employed	253 (57.8%)
Housewife	70 (16.0%)
Student	95 (21.7%)
Retired and Unemployed	20 (4.6%)
Crowding Index	
<2	137 (31.3%)
2–3	203 (46.4%)
>3	98 (22.4%)
Family monthly income	
≤30,000	304 (69.4)
30,001–100,000	122 (27.4)
>100,000	8 (1.8)
Don’t want to answer	4 (0.9)
Mean (±SD), *n* (%)	

**Table 2:** General awareness about *Aedes* mosquitoes and the surrounding physical environment of the participants, protective measures, and disease history (*n* = 438).

General Awareness, Protective Measures, and Disease History	*n* (%)
Mosquito Responsible for Dengue, Zika, Chikungunya	
*Aedes aegypti*	178 (40.6%)
All kind	3 (0.7%)
Don’t know	257 (58.7%)
*Aedes aegypti* Mosquito biting time	
Sunrise/sunset	43 (9.8%)
Daytime	134 (30.6%)
Night	113 (25.8%)
Afternoon	25 (5.7%)
All the time	43 (9.8%)
Don’t know	80 (18.3%)
Usual breeding place of *Aedes aegypti* mosquito	
Clean water	58 (13.2%)
Wastewater	334 (76.3%)
Stagnant water	1 (0.2%)
Any water	1 (0.2%)
Dense vegetation	1 (0.2%)
Moist Environment	1 (0.2%)
Don’t know	42 (9.6%)
Open drains nearby (*n* = 430)	132 (30.7%)
Deep vegetation or park nearby (*n* = 434)	94 (21.7%)
Garden in the house or neighbourhood	118 (26.9%)
Sanitary latrine in household	430 (98.2%)
Construction sites (buildings) nearby (*n* = 431)	152 (35.3%)
Road or bridge construction or repair nearby (*n* = 433)	104 (24.0%)
Number of construction sites nearby	
0	244 (55.71%)
1	110 (25.11%)
2	44 (10.05%)
>2	40 (9.13%)
Presence of items nearby that are potential *Aedes* mosquito breeding places	104 (23.7%)
Recent cleanliness or drainage work by authorities	432 (98.6%)
Recent cleanliness (Multiple response)	
By fogging	385 (87.9%)
By cleaning drains	82 (18.7%)
By removing stagnant water	39 (8.9%)
By waste management	203 (46.3%)
Personal protective measure against mosquito bite (Multiple response)	
Mosquito net	371 (84.7%)
Coil	327 (74.7%)
Closing door and window	88 (20.1%)
Spray	55 (12.6%)
Cleanliness	49 (11.2%)
Others (Repellent, Long cloth, Smoke, Fan, Mosquito bat)	16 (3.6%)
Nothing	5 (1.1%)
Travel outside Dhaka in the last 14 days	53 (12.1%)
Past diagnosed history (Multiple Responses)	
Dengue	25 (5.7%)
Chikungunya	6 (1.4%)
Zika	0 (0.0%)
No history	409 (93.4%)
Present Symptoms (Multiple Responses)	
Fatigue	420 (95.9%)
Headache	382 (87.2%)
Fever	380 (86.2%)
Arthralgia and myalgia	333 (76.0%)
Nausea	240 (54.8%)
Conjunctivitis	74 (16.9%)
Rash	11 (2.5%)
Edema	4 (0.9%)
Source of health education and information (Multiple Responses)	
Health Worker	184 (42%)
Government campaign	109 (24.9%)
Educational institute	75 (17.1%)
Media	291 (66.4%)
Family and friends	318 (72.6%)

**Table 3:** Enzyme-linked immunosorbent assay (ELISA) results of Dengue, Zika, and Chikungunya.

Virus	Antibody Type	Tested (*n*)	Positive Reactivity (*n*)	Positive Reactivity % Among Total Samples (*n* = 438)
Dengue	IgG	170	147	33.6%
	IgM	170	68	15.5%
Zika	IgG	102	92	21.0%
	IgM	102	1	0.2%
Chikungunya	IgG	168	3	0.7%

Footnotes: Of the 178 RDT-positive samples selected for ELISA testing, 8 samples were lost and could not be tested. ELISA results are therefore based on 170 available samples. Percentages are shown relative to the total study population (*n* = 438).

**Table 4:** Combined distribution of arboviral ELISA reactivity results (IgM and/or IgG) among febrile patients (*n* = 438).

Serological Category	Definition	*n*	% of Total Study Population
Non-reactive for all viruses	Non-reactive for both IgM and IgG to DENV, CHIKV, and ZIKV	275	62.8%
Reactive for DENV only	IgM and/or IgG reactive for DENV; non-reactive for CHIKV and ZIKV	70	15.9%
Reactive for CHIKV only	IgG reactive for CHIKV; non-reactive for DENV and ZIKV	0 *	0.0%
Reactive for ZIKV only	IgM and/or IgG reactive for ZIKV; non-reactive for DENV and CHIKV	6	1.4%
Reactive for DENV + ZIKV	IgM and/or IgG reactive for both DENV and ZIKV; non-reactive for CHIKV	84	19.2%
Reactive for DENV + CHIKV	IgM and/or IgG reactive for DENV and CHIKV; non-reactive for ZIKV	1	0.2%
Reactive for ZIKV + CHIKV	IgM and/or IgG reactive for ZIKV and CHIKV; non-reactive for DENV	0	0.0%
Reactive for Triple-virus	IgM and/or IgG reactive for DENV, CHIKV, and ZIKV	2	0.5%
Total		438	100%

Footnotes: * CHIKV-only reactivity was zero, but CHIKV antibodies were detected in combination with DENV and ZIKV antibodies in two participants, resulting in triple-positivity and detected in combination with DENV antibodies in one participant, resulting in dual-positivity. Percentages calculated from total study population (*n* = 438). ELISA results are suggestive, not virus-specific confirmation.

**Table 5:** Association between DENV IgG antibody reactivity and participant characteristics.

Characteristic	N	Reactive N = 147 ^1^	Non-Reactive N = 291 ^1^	*p*-Value
Age in years	438	31.5 (±14.37)	29.2 (±13.01)	0.095 ^2^
Crowding in household	438	2.8 (±2.01)	2.5 (±1.77)	0.213 ^2^
Sex	438			0.886 ^3^
Male		99 (67.35%)	194 (66.67%)	
Female		48 (32.65%)	97 (33.33%)	
Education	438			0.051 ^3^
No formal education		16 (10.88%)	36 (12.37%)	
Primary incomplete		33 (22.45%)	40 (13.75%)	
Primary complete		45 (30.61%)	75 (25.77%)	
Secondary complete		20 (13.61%)	40 (13.75%)	
Higher secondary complete		14 (9.52%)	32 (11.00%)	
Graduation or above		19 (12.93%)	68 (23.37%)	
Open drain in neighborhood	430	42 (29.17%)	90 (31.47%)	0.625 ^3^
Deep vegetation or park in neighborhood	434	30 (20.55%)	64 (22.22%)	0.689 ^3^
Garden in house or neighborhood	438	39 (26.53%)	79 (27.15%)	0.891 ^3^
Building construction site nearby	431	49 (34.03%)	103 (35.89%)	0.703 ^3^
Road, bridge construction nearby	433	31 (21.53%)	73 (25.26%)	0.392 ^3^
Potential mosquito breeding place nearby	438	38 (25.85%)	66 (22.68%)	0.462 ^3^
Recent cleanliness and drainage work (gov or others)	438	145 (98.64%)	287 (98.63%)	>0.999 ^4^
Personal protective measure against mosquito bite				
Spray	438	23 (15.65%)	32 (11.00%)	0.166 ^3^
Coil	438	107 (72.79%)	220 (75.60%)	0.523 ^3^
Repellent	438	1 (0.68%)	3 (1.03%)	>0.999 ^4^
Closing doors and windows	438	26 (17.69%)	62 (21.31%)	0.372 ^3^
Cleanliness	438	15 (10.20%)	34 (11.68%)	0.643 ^3^
Mosquito net	438	124 (84.35%)	247 (84.88%)	0.885 ^3^
Long cloths	438	1 (0.68%)	1 (0.34%)	>0.999 ^4^
Nothing	438	1 (0.68%)	4 (1.37%)	0.668 ^4^
Travel outside Dhaka	438	17 (11.56%)	36 (12.37%)	0.807 ^3^

^1^ Mean (±SD); *n* (%); ^2^ Two Sample *t*-test; ^3^ Pearson’s Chi-squared test; ^4^ Fisher’s exact test.

**Table 6:** Association between DENV IgM antibody reactivity and participant characteristics.

Characteristic	N	Reactive N = 68 ^1^	Non-Reactive N = 370 ^1^	*p*-Value
Age in years	438	28.5 (±12.37)	30.3 (±13.70)	0.331 ^2^
Crowding in household	438	3.0 (±2.52)	2.6 (±1.71)	0.078 ^2^
Sex	438			0.676 ^3^
Male		44 (64.71%)	249 (67.30%)	
Female		24 (35.29%)	121 (32.70%)	
Education	438			0.022 ^3^
No formal education		6 (8.82%)	46 (12.43%)	
Primary incomplete		18 (26.47%)	55 (14.86%)	
Primary complete		20 (29.41%)	100 (27.03%)	
Secondary complete		9 (13.24%)	51 (13.78%)	
Higher secondary complete		10 (14.71%)	36 (9.73%)	
Graduation or above		5 (7.35%)	82 (22.16%)	
Open drain in neighborhood	430	23 (34.33%)	109 (30.03%)	0.483 ^3^
Deep vegetation or park in neighborhood	434	14 (20.59%)	80 (21.86%)	0.815 ^3^
Garden in house or neighborhood	438	16 (23.53%)	102 (27.57%)	0.490 ^3^
Building construction site nearby	431	27 (41.54%)	125 (34.15%)	0.251 ^3^
Road, bridge construction nearby	433	15 (23.08%)	89 (24.18%)	0.847 ^3^
Potential mosquito breeding place nearby	438	19 (27.94%)	85 (22.97%)	0.376 ^3^
Recent cleanliness and drainage work (gov or others)	438	66 (97.06%)	366 (98.92%)	0.235 ^4^
Personal protective measure against mosquito bite				
Spray	438	8 (11.76%)	47 (12.70%)	0.830 ^3^
Coil	438	53 (77.94%)	274 (74.05%)	0.498 ^3^
Repellent	438	0 (0.00%)	4 (1.08%)	>0.999 ^4^
Closing doors and windows	438	12 (17.65%)	76 (20.54%)	0.584 ^3^
Cleanliness	438	7 (10.29%)	42 (11.35%)	0.799 ^3^
Mosquito net	438	60 (88.24%)	311 (84.05%)	0.379 ^3^
Long cloths	438	0 (0.00%)	2 (0.54%)	>0.999 ^4^
Nothing	438	0 (0.00%)	5 (1.35%)	>0.999 ^4^
Travel outside Dhaka	438	9 (13.24%)	44 (11.89%)	0.755 ^3^

^1^ Mean (±SD); *n* (%); ^2^ Two Sample *t*-test; ^3^ Pearson’s Chi-squared test; ^4^ Fisher’s exact test.

**Table 7:** Association between ZIKV IgG antibody reactivity and participant characteristics.

Characteristic	N	Reactive N = 92 ^1^	Non-Reactive N = 346 ^1^	*p*-Value
Age in years	438	33.5 (±14.99)	29.1 (±12.95)	0.005 ^2^
Crowding in household	438	2.6 (±1.39)	2.6 (±1.96)	0.678 ^2^
Sex	438			0.909 ^3^
Male		62 (67.39%)	231 (66.76%)	
Female		30 (32.61%)	115 (33.24%)	
Education	438			0.949 ^3^
No formal education		11 (11.96%)	41 (11.85%)	
Primary incomplete		15 (16.30%)	58 (16.76%)	
Primary complete		29 (31.52%)	91 (26.30%)	
Secondary complete		11 (11.96%)	49 (14.16%)	
Higher secondary complete		9 (9.78%)	37 (10.69%)	
Graduation or above		17 (18.48%)	70 (20.23%)	
Open drain in your area	430	25 (27.78%)	107 (31.47%)	0.499 ^3^
Deep vegetation or park in neighborhood	434	20 (21.98%)	74 (21.57%)	0.934 ^3^
Garden in house or neighborhood	438	26 (28.26%)	92 (26.59%)	0.748 ^3^
Building construction site nearby	431	26 (28.57%)	126 (37.06%)	0.132 ^3^
Road, bridge construction nearby	433	20 (21.74%)	84 (24.63%)	0.564 ^3^
Potential mosquito breeding place nearby	438	21 (22.83%)	83 (23.99%)	0.816 ^3^
Recent cleanliness and drainage work (gov or others)	438	90 (97.83%)	342 (98.84%)	0.610 ^4^
Personal protective measure against mosquito bite				
Spray	438	15 (16.30%)	40 (11.56%)	0.222 ^3^
Coil	438	64 (69.57%)	263 (76.01%)	0.206 ^3^
Repellent	438	2 (2.17%)	2 (0.58%)	0.196 ^4^
Closing doors and windows	438	16 (17.39%)	72 (20.81%)	0.467 ^3^
Cleanliness	438	11 (11.96%)	38 (10.98%)	0.792 ^3^
Mosquito net	438	77 (83.70%)	294 (84.97%)	0.763 ^3^
Long cloths	438	0 (0.00%)	2 (0.58%)	>0.999 ^4^
Nothing	438	1 (1.09%)	4 (1.16%)	>0.999 ^4^
Travel outside Dhaka	438	11 (11.96%)	42 (12.14%)	0.962 ^3^

^1^ Mean (±SD); *n* (%); ^2^ Two Sample *t*-test; ^3^ Pearson’s Chi-squared test; ^4^ Fisher’s exact test.

**Table 8:** Association between overall arboviral antibody reactivity and other factors (*n*= 438).

Characteristic	N	Reactive N = 163 ^1^	Non-Reactive N = 275 ^1^	*p*-Value
Age in years	438	31.1 (±14.13)	29.3 (±13.11)	0.184 ^2^
Crowding in household	438	2.7 (±1.94)	2.6 (±1.81)	0.371 ^2^
Sex	438			0.827 ^3^
Male		108 (66.26%)	185 (67.27%)	
Female		55 (33.74%)	90 (32.73%)	
Education	438			0.168 ^3^
No formal education		17 (10.43%)	35 (12.73%)	
Primary incomplete		33 (20.25%)	40 (14.55%)	
Primary complete		49 (30.06%)	71 (25.82%)	
Secondary complete		22 (13.50%)	38 (13.82%)	
Higher secondary complete		19 (11.66%)	27 (9.82%)	
Graduation or above		23 (14.11%)	64 (23.27%)	
Open drain in your area	430	48 (30.00%)	84 (31.11%)	0.809 ^3^
Deep vegetation or park in neighborhood	434	34 (20.99%)	60 (22.06%)	0.793 ^3^
Garden in house or neighborhood	438	43 (26.38%)	75 (27.27%)	0.839 ^3^
Building construction site nearby	431	54 (33.96%)	98 (36.03%)	0.665 ^3^
Road, bridge construction nearby	433	36 (22.50%)	68 (24.91%)	0.571 ^3^
Potential mosquito breeding place nearby	438	41 (25.15%)	63 (22.91%)	0.594 ^3^
Recent cleanliness and drainage work (gov or others)	438	159 (97.55%)	273 (99.27%)	0.201 ^4^
Personal protective measure against mosquito bite				
Spray	438	24 (14.72%)	31 (11.27%)	0.292 ^3^
Coil	438	118 (72.39%)	209 (76.00%)	0.401 ^3^
Repellent	438	2 (1.23%)	2 (0.73%)	0.631 ^4^
Closing doors and windows	438	30 (18.40%)	58 (21.09%)	0.498 ^3^
Cleanliness	438	18 (11.04%)	31 (11.27%)	0.941 ^3^
Mosquito net	438	139 (85.28%)	232 (84.36%)	0.798 ^3^
Long cloths	438	1 (0.61%)	1 (0.36%)	>0.999 ^4^
Nothing	438	1 (0.61%)	4 (1.45%)	0.655 ^4^
Travel outside Dhaka	438	20 (12.27%)	33 (12.00%)	0.933 ^3^

^1^ Mean (±SD); *n* (%); ^2^ Two Sample *t*-test; ^3^ Pearson’s Chi-squared test; ^4^ Fisher’s exact test.

## Data Availability

Due to icddr,b’s data access policy regarding participant-identifying information, the data are available upon request from the Research & Clinical Administration and Strategy (RCAS) at icddr,b.

## Abbreviations

The following abbreviations are used in this manuscript:

ELISA	Enzyme-linked immunosorbent assay
CHIKV	Chikungunya virus
DENV	Dengue virus
ZIKV	Zika virus
PRNT	Plaque reduction neutralization test
RDT	Rapid diagnostic test
